# Ablation of TRPV1 Abolishes Salicylate-Induced Sympathetic Activity Suppression and Exacerbates Salicylate-Induced Renal Dysfunction in Diet-Induced Obesity

**DOI:** 10.3390/cells10051234

**Published:** 2021-05-18

**Authors:** Beihua Zhong, Shuangtao Ma, Donna H. Wang

**Affiliations:** 1 Division of Nanomedicine and Molecular Intervention, Department of Medicine, Michigan State University, East Lansing, MI 48824, USA; beihuazhong@mail.com (B.Z.); mashuang@msu.edu (S.M.); 2 Neuroscience Program, Michigan State University, East Lansing, MI 48824, USA; 3 Cell and Molecular Biology Program, Michigan State University, East Lansing, MI 48824, USA

**Keywords:** TRPV1, obesity, sodium salicylate, afferent renal nerve activity, renal dysfunction, blood pressure

## Abstract

Sodium salicylate (SA), a cyclooxygenase inhibitor, has been shown to increase insulin sensitivity and to suppress inflammation in obese patients and animal models. Transient receptor potential vanilloid 1 (TRPV1) is a nonselective cation channel expressed in afferent nerve fibers. Cyclooxygenase-derived prostaglandins are involved in the activation and sensitization of TRPV1. This study tested whether the metabolic and renal effects of SA were mediated by the TRPV1 channel. Wild-type (WT) and TRPV1^−/−^ mice were fed a Western diet (WD) for 4 months and received SA infusion (120mg/kg/day) or vehicle for the last 4 weeks of WD feeding. SA treatment significantly increased blood pressure in WD-fed TRPV1^−/−^ mice (*p* < 0.05) but not in WD-fed WT mice. Similarly, SA impaired renal blood flow in TRPV1^−/−^ mice (*p* < 0.05) but not in WT mice. SA improved insulin and glucose tolerance in both WT and TRPV1^−/−^ mice on WD (all *p* < 0.05). In addition, SA reduced renal p65 and urinary prostaglandin E2, prostaglandin F1α, and interleukin-6 in both WT and TRPV1^−/−^ mice (all *p* < 0.05). SA decreased urine noradrenaline levels, increased afferent renal nerve activity, and improved baroreflex sensitivity in WT mice (all *p* < 0.05) but not in TRPV1^−/−^ mice. Importantly, SA increased serum creatinine and urine kidney injury molecule-1 levels and decreased the glomerular filtration rate in obese WT mice (all *p* < 0.05), and these detrimental effects were significantly exacerbated in obese TRPV1^−/−^ mice (all *p* < 0.05). Lastly, SA treatment increased urine albumin levels in TRPV1^−/−^ mice (*p* < 0.05) but not in WT mice. Taken together, SA-elicited metabolic benefits and anti-inflammatory effects are independent of TRPV1, while SA-induced sympathetic suppression is dependent on TRPV1 channels. SA-induced renal dysfunction is dependent on intact TRPV1 channels. These findings suggest that SA needs to be cautiously used in patients with obesity or diabetes, as SA-induced renal dysfunction may be exacerbated due to impaired TRPV1 in obese and diabetic patients.

## 1. Introduction

Cyclooxygenase 2 (COX-2) contributes to the development of obesity and obesity-associated metabolic syndrome [1]. COX-2 is considered to be an inducible enzyme and is upregulated in inflammatory conditions [2,3]. Type 2 diabetes is related to COX-2-mediated inflammation [4], and inhibition of COX-2 in the diabetic rat confers renal protection [5]. Sodium salicylate (SA), a COX inhibitor, inhibits inflammation and improves insulin sensitivity and hyperleptinemia in obesity. On the other hand, COX-2-derived prostacyclin (PGI2) and prostaglandin E2 (PGE2) exert a natriuretic effect and increase renal blood flow and glomerular filtration rate (GFR) [6,7]. Renal COX-mediated generation of prostanoids is enhanced in metabolic disorders, which may play a counter-regulatory role against renal vasoconstriction induced by elevated renal sympathetic nerve activity (RSNA) in obesity. In addition, PGI2 is a potent vasodilator that decreases peripheral vascular resistance during the initial phase of renal hypertension [8,9,10,11]. Therefore, inhibiting COX-2 with SA could improve insulin resistance at the cost of renal function deterioration.

Understanding the action of mechanisms of COX-2 inhibitors may facilitate the development of a better COX-2-targeting treatment without the adverse effects on renal function. The transient receptor potential subfamily V member 1 (TRPV1) cation channel is expressed in renal afferent nerve fibers [12]. Activation of TRPV1 in the renal pelvis leads to an increase in ipsilateral renal afferent nerve activity (ARNA) [9,10,11] and a long-lasting depression of efferent RSNA [13]. TRPV1 activation enhances ARNA by inducing the release of substance P (SP) from sensory nerves [11]. Increased ARNA provides an important contribution to the maintenance of low RSNA, which is essential in preventing renal sodium retention as well as the regulation of arterial pressure [11,13]. The functions of TRPV1 and ARNA are impaired in obesity [14]. Obesity is associated with an increase in RSNA [15,16] and impaired ARNA and baroreflex sensitivity (BRS) [17]. In addition, lack of TRPV1 exacerbates obesity [18]. Evidence suggests that there may be connections between COX-2 and TRPV1 [19]. It was reported that COX-2-derived PGE2 increased the release of substance P (SP) from renal pelvic sensory nerves and enhanced ARNA [20]. Therefore, the effects of COX-2 inhibitors may be mediated by TRPV1. However, it is unknown whether the metabolic and renal effects of COX-2 inhibitors are dependent on TRPV1 channels.

In the present study, we investigated whether the TRPV1 channel was involved in the metabolic and renal effects of SA treatment in diet-induced obese mice using TRPV1 gene knockout mice.

## 2. Materials and Methods

### 2.1. Animals

All experimental procedures involving animals were approved by the Institutional Animal Care and Use Committee of Michigan State University (08-17-148-00). This study followed the rules of the Declaration of Helsinki. The male TRPV1 gene knockout (TRPV1^−/−^) mice (B6.129S4-TRPV1^tm1Jul^, Stock No: 003770) and matching control wild-type (WT) C57BL/6J mice (Jackson Laboratory, Bar Harbor, Maine) were used. The TRPV1^−/−^ mice had been backcrossed to C57BL/6J mice for 10 generations. Three-week-old TRPV1^−/−^ (*n* = 14) and WT (*n* = 14) mice were fed a Western diet (WD, protein 17.3%, carbohydrate 48.5%, fat 21.2% (mainly from milkfat), 42% kcal from fat; 88137, Harlan Teklad) for 4 months, during which the mice had free access to tap water. The mice were maintained with a normal 12 h/12 h light/dark cycle. Room temperature was maintained at 23.0 ± 1.0 °C, with a relative humidity between 40 and 60%.The mice (*n* = 7 in each group) received either sodium salicylate (SA, 120 mg/kg body weight per day, administered by Alzet pumps, Model 2004, 1 mL, Alzet Corporation, Palo Alto, CA, USA) or vehicle (saline, administered by the same Alzet pumps) for 4 weeks during the last 4 weeks of diet intervention. SA was purchased from Sigma-Aldrich (#S3007, St. Louis, MO, USA) and freshly dissolved in saline before use.

### 2.2. Telemetry Blood Pressure Assay

Mean arterial pressure (MAP) and heart rate (HR) of the mice (*n* = 6 in each group) were determined using a telemetry system (Data Sciences International, St. Paul, MN, USA). In brief, the mice, after diet and SA treatment, were anesthetized with ketamine and xylazine (80 and 5 mg/kg, i.p., respectively), and then the transmitter catheter was implanted into the left carotid artery. Mice were allowed to recovery for 7 days before ambulatory 24 h MAP was recorded.

### 2.3. Glucose and Insulin Tolerance Testing and Serum Leptin and Insulin Measurement

At the end of the 16-week treatment, mice (*n* = 5–7 in each group) were fasted for 10 h and then given glucose (2 g/kg body weight, i.p.). Tail vein blood was sampled for glucose determination using an Accu-Chek glucose meter (Roche Diagnostics, Indianapolis, IN, USA) at 0, 30, 60, 90, and 120 min after glucose administration. The areas under the curve (AUC) for glucose were calculated according to the trapezoidal rule. After 48 h, mice were fasted for 6 h, and then insulin (0.75 IU/kg body weight diluted in 0.9% saline) was injected intraperitoneally, and subsequent blood samples were taken from the tail tip. Glucose was quantified at 0, 30, 60, and 120 min after insulin administration using an Accu-Chek glucose meter (Roche Diagnostics). The AUC vs. time curve was calculated with the trapezoidal rule. Tail vein blood was also collected at baseline, and serum was immediately separated and stored at −80 °C. Insulin and leptin levels were measured using a commercial kit (Crystal Chem Inc., Downers Grove, IL, USA).

### 2.4. Recording of Afferent Renal Nerve Activity

After the mice (*n* = 5–7 in each group) were anesthetized with ketamine and xylazine (80 and 5 mg/kg, i.p., respectively), the renal nerves were isolated at the angle between the abdominal aorta and the renal artery via a left flank incision [12,21]. The nerves were placed on the bipolar, stainless-steel electrode to record multifiber nerve activity. The electrode was connected to a high-impedance probe (HIP-511, Grass Instruments). The signals were amplified 20,000×, filtered with a high-frequency cutoff at 1000 Hz and a low-frequency cutoff at 100 Hz by a Grass model P511 AC Amplifier and recorded by a Gould 2400 s recorder (Gould Instrument System, Valley View, OH, USA). After the renal nerve activity was verified using its pulse synchronous rhythmicity with the heartbeat, the nerves were sectioned, and ARNA was recorded from the distal cut end of a renal nerve branch. The electrode was fixed to the renal nerve with Kwik-Cast & Kwik-Sil (World Precision Instruments, Sarasota, FL, USA). The experiment started after the placement of nerve electrodes and physiological stabilization for 60 min. Two MD-2000 microdialysis tubes (ID 0.18/OD 0.22 mm; BASi) were bonded together and placed inside the left ureter via a midline incision. One of the tubes, of which the tip extended 1 to 2 mm further into the renal pelvis compared with the other, was used for drug perfusion, whereas the other was used for urine draining. The perfusion was performed at a rate of 10 μL/min. Capsaicin (10^−6^ mmol/L) was perfused into the renal pelvis in 3-min periods, and the recovery value of renal nerve activity was recorded 10 min after the treatment. The postmortem renal nerve activity recorded as the background signal of renal nerve activity was subtracted from all of the nerve activity recording values. Capsaicin-stimulated renal nerve activity was expressed as a percentage of its baseline value prior to the application of capsaicin.

### 2.5. Determination of Baroreflex Function

The baroreflex function of the mice (*n* = 5–7 in each group) was evaluated by measuring the reflex changes in RSNA and HR in response to decreases and increases in MAP induced by intravenous infusion of 50 μg/mL sodium nitroprusside (SNP) and 125 μg/mL phenylephrine (PE), respectively. SNP and PE were administered via the jugular vein in successive ramped infusions at an initial rate of 5 μL/min, increased by 5 μL/min every 30 s [22]. The PE and SNP infusions were done separately, with one drug administered after the blood pressure response to the other drug had returned to baseline level, and the order of drugs was administered randomly. Infusions were stopped if MAP reached a minimum of 60 mmHg or a maximum of 140 mmHg. The RSNA was recorded from the proximal cut end of a renal nerve and hooked up to electrodes. The postmortem renal nerve activity recorded as the background of renal nerve activity was subtracted from all of the values. Baroreflex-mediated changes in RSNA were expressed as a percentage of its baseline value. Baroreflex modulation of RSNA and HR was estimated by calculating (1) the percent change in integrated activity and (2) the change in HR in relation to the change in mean BP induced by PE and SNP.

### 2.6. Renal Blood Flow Recording

Anesthesia was induced with 5% isoflurane (Abbott Laboratories, Chicago, IL, USA) and maintained with 1% isoflurane, and the mice (*n* = 5–7 in each group) were placed on a heating pad to maintain body temperature at 37 °C [23,24]. After surgical preparation, the kidney was placed in a clay cup without exerting tension on the renal vessels. The left jugular vein was cannulated for injections and infusion of a 0.9% sodium chloride solution containing 1% bovine serum albumin at a rate of 0.2 mL/Kg/min throughout the experiment. Cortical and medullary blood flows (CBF and MBF, respectively) were measured simultaneously by a dual-channel, laser-Doppler flowmeter (Periflux 5000, Perimed, North Royalton, OH, USA). For measurement of CBF, the probe was placed perpendicular to the surface of the cortex and MBF was measured by a probe inserted into the outer medulla at a depth of 3–4 mm. The position of the probe in the outer medulla was verified at the end of each experiment by dissection of the kidney. The experiment started after the placement of the probe and physiological stabilization for 60 min to obtain baseline recordings of CBF and MBF. Next, the mice received intravenous bolus (10 µL) injections of angiotensin II (Ang II, Sigma) at doses of 0.5, 2.5, and 12.5 ng/kg. Consecutive administrations of Ang II were separated by a period of 10 min to allow a full recovery of hemodynamic variables. Electrical signals of both probes were digitized and recorded in real time and analyzed by Perisoft for Windows software (Perimed).

### 2.7. Renal NF-κB p65 Activity Assay

Nuclear protein was extracted from the kidney with a nuclear extract kit (Active Motif, Carlsbad, CA, USA) based on the manufacturer’s instructions. The binding activities of free NF-κB p65 in nuclear extracts were determined with the use of the TransAM NF-κB p65 assay kit (Active Motif) following the manufacturer’s protocol. The plate was read at 450 nm using an absorbance microplate reader (Molecular Devices, Sunnyvale, CA, USA).

### 2.8. Plasma and Urine Analysis

At the end of the 16-week treatment, mice were placed in mouse metabolic cages for 24 h urine collection. Urine samples were centrifuged and stored at −80 °C. Urinary albumin and noradrenaline were measured with enzyme-linked immunosorbent assay (ELISA) kits (Mouse Albumin ELISA, #1011, Exocell, Philadelphia, PA, USA; Norepinephrine ELISA Kit, #E4360, Biovision, Milpitas, CA, USA). Urinary kidney injury molecule-1 (KIM-1) as a marker of renal injury was quantified using a mouse kim-1 Elisa kit (CL0880, Cell applications Inc, CA, USA) according to the manufacturer’s protocol. Plasma creatinine concentrations were assayed using the Creatinine Colorimetric/Fluorometric Assay Kit (K625, Biovision, Milpitas, CA, USA). Endogenous creatinine clearance is a sensitive and accurate method for assessing glomerular filtration rate (GFR). Plasma and urine creatinine levels were determined using the above-mentioned assay kit (K625, Biovision) and calculation of creatinine clearance: GFR = U[Cr] × Volume]/P[Cr] × [Time] [25]. Since the half-life of PGI2 was short, 6-keto-PGFla, its stable and inactive metabolite, was measured. A PGE2 EIA Kit-Monoclonal (#514010, Cayman Chemical Company, MI, USA) and a 6-keto-PGFla EIA Kit (#515211, Cayman Chemical Company, MI, USA) were used according to the manufacturer’s instructions.

### 2.9. Statistical Analysis

All values are expressed as mean ± SEM. Differences among groups were performed by two-way ANOVA analysis followed by the Tukey–Kramer multiple comparison test. The results were considered statistically significant at *p* < 0.05.

## 3. Results

### 3.1. Effects of SA on Blood Pressure

**Figure 1:**
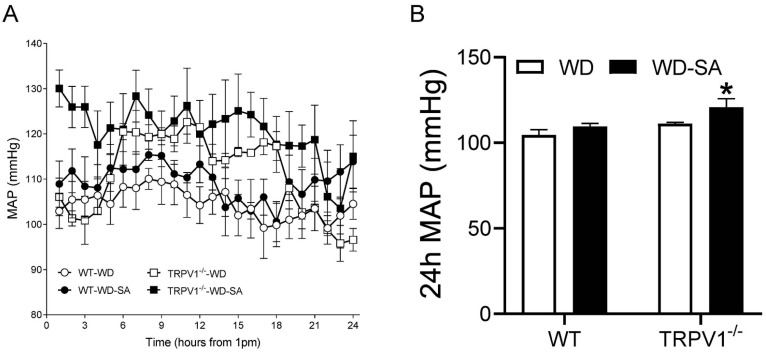
SA treatment-induced hypertension in obese TRPV1^−/−^ mice. Telemetric recording of ambulatory blood pressure (**A**) and 24-h mean arterial pressure (MAP) (**B**) in WD-fed WT mice and TRPV1^−/−^ mice treated with or without SA. Differences among groups were performed by two-way ANOVA analysis followed by the Tukey–Kramer multiple comparison test. Values are mean ± SEM; *n* = 5–6; * *p* < 0.05 vs. TRPV1^−/−^ mice treated without SA.

Ambulatory blood pressure was measured by telemetry after the mice had been fed with WD for 16 weeks and treated with SA or vehicle for 4 weeks (Figure 1A). SA treatment did not change the 24 h MAP of WD-fed WT mice but significantly increased MAP in WD-fed TRPV1^−/−^ mice compared with WD-fed TRPV1^−/−^ mice treated without SA (*p* < 0.05, Figure 1B). SA treatment had no significant effects on body weight of either WT or TRPV1^−/−^ mice (WT-WD, 47.1 ± 3.4 g; WT-WD-SA, 41.6 ± 3.2 g; TRPV1^−/−^-WD, 46.2 ± 3.7 g; TRPV1^−/−^-WD-SA, 46.0 ± 2.9 g).

### 3.2. Effects of SA on Glucose Tolerance and Insulin Resistance

**Figure 2:**
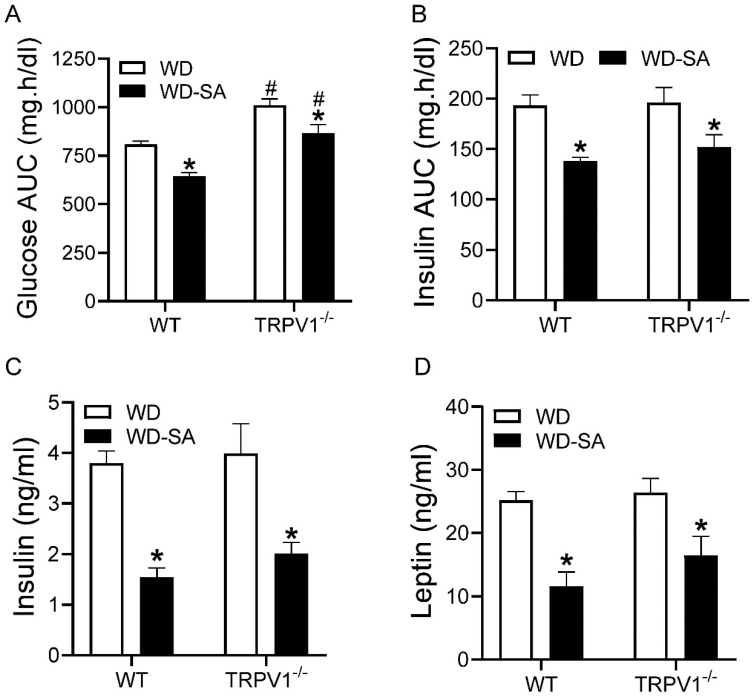
Effects of SA on glucose tolerance and insulin resistance. Area under the curve (AUC) of the insulin tolerance test (**A**) and glucose tolerance test (**B**) in WD-fed WT mice and TRPV1^−/−^ mice treated with or without SA. Plasma insulin (**C**) and leptin (**D**) levels of WD-fed WT mice and TRPV1^−/−^ mice treated with or without SA. Differences among groups were performed by two-way ANOVA analysis followed by the Tukey–Kramer multiple comparison test. Values are mean ± SEM; *n* = 6–7; * *p* < 0.05 vs. isogenic mice treated without SA; ^#^ *p* < 0.05 vs. WT mice with the same treatment.

Glucose tolerance, reflected as the AUC of the glucose tolerance test, was significantly improved by treatment with SA in both WT and TRPV1^−/−^ mice (Figure 2A, both *p* < 0.05). Moreover, glucose tolerance was impaired in TRPV1^−/−^ mice compared with WT mice, independent of SA treatment (Figure 2A, both *p* < 0.05). The AUC for the insulin tolerance test was reduced in the SA-treated groups for both WT and TRPV1^−/−^ mice (Figure 2B, both *p* < 0.05). Fasting insulin and leptin concentrations were lower in both strains with SA vs. vehicle treatment (Figure 2C,D, all *p* < 0.05). There were no differences in insulin and leptin levels between WT and TRPV1^−/−^ mice despite treatment with SA.

### 3.3. Effects of SA on Autonomic Nerve Activity

**Figure 3:**
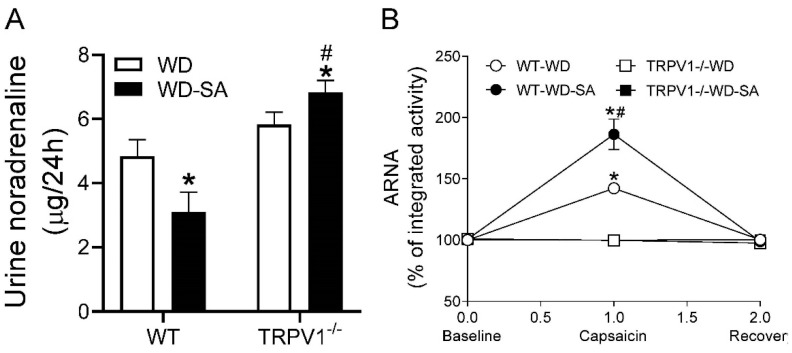
Effects of SA on autonomic nerve activity. (**A**) Urinary norepinephrine levels of WD-fed WT mice and TRPV1^−/−^ mice treated with or without SA. (**B**) Intra-renal pelvic infusion of capsaicin-induced afferent renal nerve activity (ARNA). Differences among groups were performed by two-way ANOVA analysis followed by the Tukey–Kramer multiple comparison test. Values are mean ± SEM; *n* = 6–7; * *p* < 0.05 vs. isogenic mice treated without SA; ^#^ *p* < 0.05 vs. WT mice with the same treatment.

SA treatment significantly decreased urinary noradrenaline in WD-fed WT mice, while it increased it in WD-fed TRPV1^−/−^ mice (Figure 3A, both *p* < 0.05). Capsaicin, a TRPV1 agonist, was perfused into the renal pelvis, which stimulated ARNA in WT mice but not in TRPV1^−/−^ mice (Figure 3B). The capsaicin-induced increase in ipsilateral ARNA was enhanced by SA treatment in WD-fed WT mice (Figure 3B, *p* < 0.05). These results suggest that SA treatment suppressed RSNA and improved ARNA, likely through TRPV1.

### 3.4. Effects of SA Treatment on Baroreflex Sensitivity

**Figure 4:**
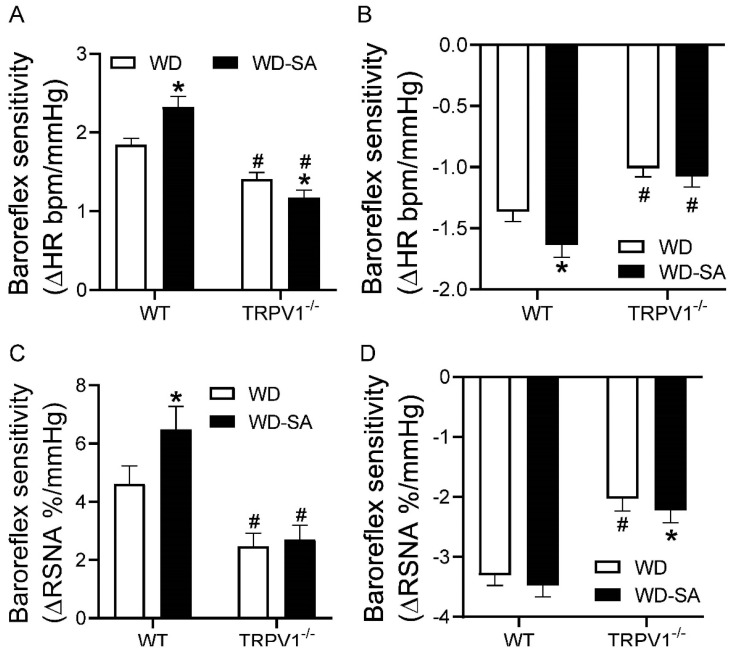
Effects of SA treatment on baroreflex sensitivity. Baroreflex sensitivity of heart rate (HR) in response to intravenous injection of sodium nitroprusside (50μg/mL) (**A**) and phenylephrine (125 μg/mL) (**B**) in WD-fed WT mice and TRPV1^−/−^ mice treated with or without SA. Baroreflex sensitivity of RSNA in response to intravenous injection of sodium nitroprusside (50 μg/mL) (**C**) and phenylephrine (125 μg/mL) (**D**) in WD-fed WT mice and TRPV1^−/−^ mice treated with or without SA. Differences among groups were performed by two-way ANOVA analysis followed by the Tukey–Kramer multiple comparison test. Values are mean ± SEM; *n* = 5–7; * *p* < 0.05 vs. isogenic mice treated without SA; ^#^ *p* < 0.05 vs. WT mice with the same treatment.

Baroreflex sensitivity was evaluated by measuring the changes of HR and RSNA in response to phenylephrine- and nitroprusside-induced increase and decrease of MAP, respectively. The baroreflex sensitivity was improved with SA vs. vehicle treatment in WD-fed WT mice, but not in WD-fed TRPV1^−/−^ mice (Figure 4). These results suggest that SA improved baroreflex sensitivity, which was dependent on intact TRPV1 channels.

### 3.5. Effects of SA on Renal Blood Flow

**Figure 5:**
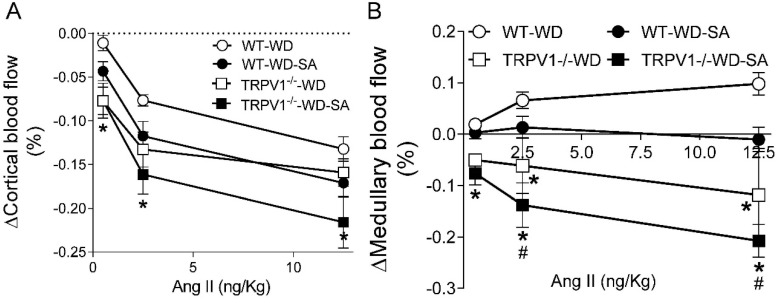
Effects of SA on renal blood flow. Peak changes of mean renal cortical blood flow (CBF) (**A**) and medullary blood flow (MBF) (**B**) in response to intravenous bolus injections of angiotensin II (Ang II) at doses of 0.5, 2.5, and 12.5 ng/kg in WD-fed WT mice and TRPV1^−/−^ mice treated with or without SA. Differences among groups were performed by two-way ANOVA analysis followed by the Tukey–Kramer multiple comparison test. Values are mean ± SEM; *n* = 5–7; * *p* < 0.05 vs. isogenic mice treated without SA; ^#^ *p* < 0.05 vs. WT mice with the same treatment.

Infusions of Ang II (0.5 to 12.5 ng/Kg, i.v.) dose-dependently reduced CBF, and a further decrease was observed in the WD-fed TRPV1^−/−^ mice compared with WD-fed WT mice (Figure 5A). SA treatment had no significant effects on CBF in either WT or TRPV1^−/−^ mice (Figure 5A). Infusions of Ang II dose-dependently increased MBF in WD-fed WT mice, while they significantly decreased MBF in WD-fed TRPV1^−/−^ mice (Figure 5B), indicating TRPV1 ablation abolished the response of CBF and MBF to Ang II infusion in mice fed WD. Moreover, SA decreased the response of MBF to Ang II infusion in WD-fed TRPV1^−/−^ mice compared to WD-fed WT mice.

### 3.6. Effects of SA on Renal Inflammatory Markers

**Figure 6:**
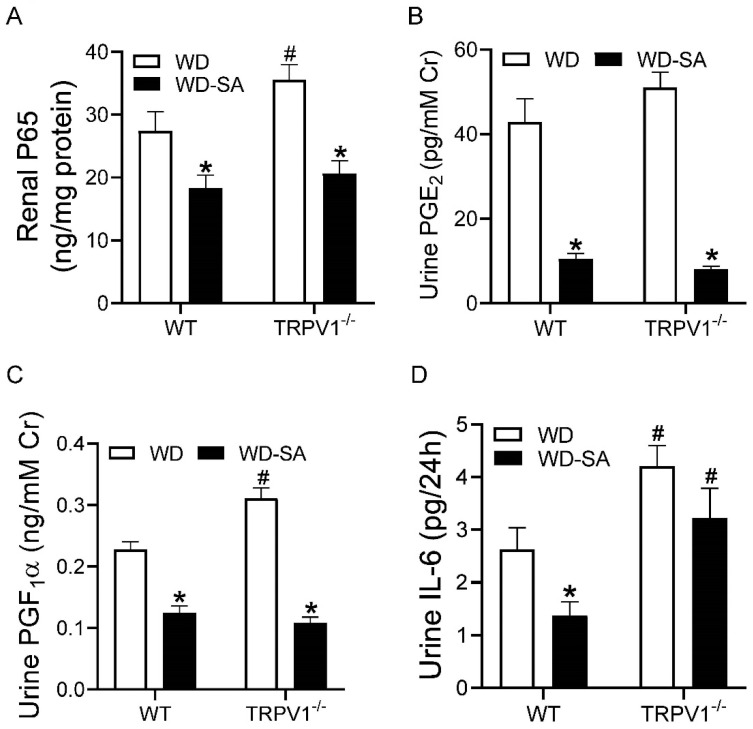
Effects of SA on renal inflammatory markers. Renal p65 binding activity (**A**), urine PGE2 (**B**), PGF1α (**C**), and IL-6 (**D**) in WD-fed WT mice and TRPV1^−/−^ mice treated with or without SA. Differences among groups were performed by two-way ANOVA analysis followed by the Tukey–Kramer multiple comparison test. Values are mean ± SEM; *n* = 5–7; * *p* < 0.05 vs. isogenic mice treated without SA; ^#^ *p* < 0.05 vs. WT mice with the same treatment.

SA treatment significantly decreased the binding activity of renal NF-κB p65 and urine PGE2 and PGF1α levels in both WD-fed WT and WD-fed TRPV1^−/−^ mice (Figure 6A–C, all *p* < 0.05). SA lowered urine Il-6 levels in WD-fed WT mice (*p* < 0.05) but not in WD-fed TRPV1^−/−^ mice (Figure 6D). In addition, WD-fed TRPV1^−/−^ mice had higher renal NF-κB p65 binding activity and urine PGF1α and IL-6 levels than WD-fed WT mice (Figure 6A,C,D, all *p* < 0.05).

### 3.7. Effects of SA on Renal Function

**Figure 7:**
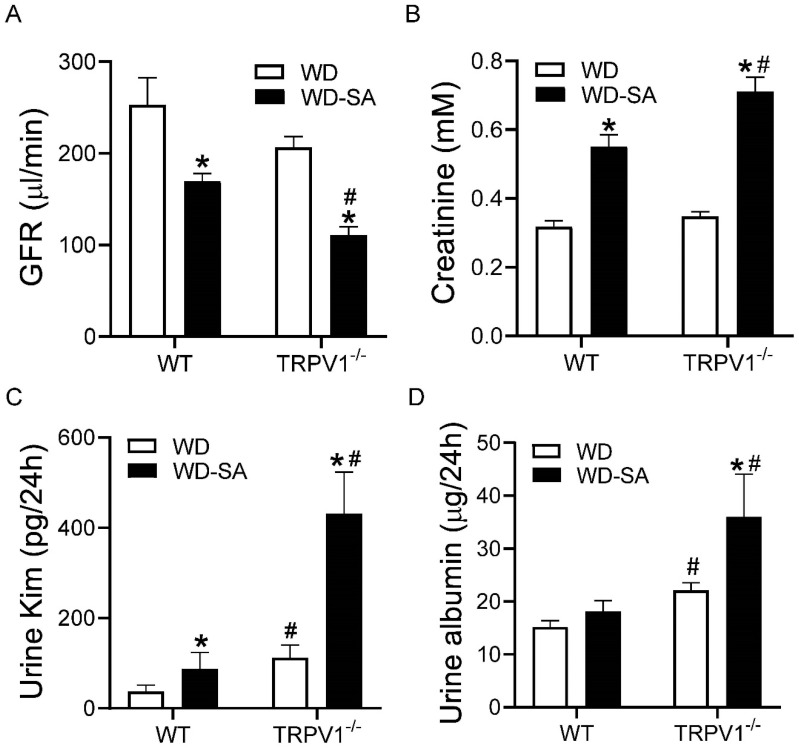
Effects of SA on renal function. GFR (**A**), serum creatinine (**B**), urine Kim-1 (**C**), and urine albumin (**D**) in WD-fed WT mice and TRPV1^−/−^ mice treated with or without SA. Differences among groups were performed by two-way ANOVA analysis followed by the Tukey–Kramer multiple comparison test. Values are mean ± SEM; *n* = 5–7; * *p* < 0.05 vs. isogenic mice treated without SA; ^#^ *p* < 0.05 vs. WT mice with the same treatment.

SA treatment decreased GFR and increased plasma creatinine and urine Kim-1 levels in both WD-fed WT and TRPV1^−/−^ mice (Figure 7A–C, all *p* < 0.05), to a greater magnitude in TRPV1^−/−^ mice than in WT mice (Figure 7A–C, all *p* < 0.05). SA increased urinary albumin levels in WD-fed TRPV1^−/−^ mice but not in WD-fed WT mice (Figure 7D, *p* < 0.05). Urinary Kim-1 and albumin levels in WD-fed TRPV1^−/−^ mice were higher than those in WT mice fed with WD (Figure 7C,D, *p* < 0.05).These results indicate that SA caused more significant renal dysfunction in TRPV1^−/−^ mice than in WT mice.

## 4. Discussion

There are three main findings in the present study. Firstly, SA treatment attenuated renal inflammation and improved insulin resistance in both obese WT and TRPV1^−/−^ mice, indicating that the beneficial effects of SA on insulin sensitivity are independent of TRPV1. Secondly, SA treatment suppressed RSNA, increased ARNA, and improved baroreflex sensitivity in obese WT mice but not in obese TRPV1^−/−^ mice, suggesting the nerve-protecting effects of SA were dependent on TRPV1. Thirdly, SA treatment caused renal dysfunction in obese WT mice, and this detrimental effect was exacerbated in TRPV1^−/−^ mice, indicating that SA-induced renal dysfunction was dependent on whether TRPV1 channels were intact.

Activation of TRPV1 expressed in afferent sensory nerve fibers leads to an increase in ARNA [10]. Increased ARNA contributes to the maintenance of a low efferent RSNA, which is essential in preventing renal sodium retention and the regulation of arterial pressure [11,13]. The function of sensory nerves, especially TRPV1-positive nerves, is impaired in obesity. Obesity decreased capsaicin-induced transmitter release from sensory nerves [14]. Previous studies showed that TRPV1 were expressed throughout the entire baroreceptive afferent pathway, and TRPV1 ablation impaired the baroreflex control of efferent RSNA and HR [22]. The baroreceptor dysfunction is an initial change in obesity, which is followed by sympathetic overactivation and hypertension [17]. TRPV1^−/−^ mice fed with WD had increased urinary Kim-1 and albumin levels compared with WD-fed WT mice, indicating a reno-protective role of TRPV1 in obese mice. The present study demonstrated that SA treatment suppressed RSNA, increased ARNA, and improved baroreflex sensitivity in obese WT mice, likely through TRPV1. Renal afferent nerves could be damaged and afferent nerve activity impaired by inflammation in obesity [26,27]. SA may increase ARNA through its anti-inflammatory effects.

SA reverses hyperglycemia, hyperinsulinemia, and hyperleptinemia in WD-induced obese mice [28,29,30,31]. The present study demonstrated that SA treatment decreased plasma insulin, glucose, and leptin levels in obese WT mice as well as in obese TRPV1^−/−^ mice. These results suggest that the metabolic benefits of SA are probably not mediated by its nerve-protective effects, as the latter is dependent on intact TRPV1 channels. Leptin is an important factor driving obesity-associated arterial hypertension. Leptin-induced increases in RSNA can be suppressed by baroreflex activation [32]. Impaired baroreflex leads to increased RSNA and hypertension in patients with type 2 diabetes [33,34,35]. Therefore, it is possible that the nerve-protective effects of SA are mediated by its effects on leptin levels. It is worth mentioning that SA may exert vasodilatory effects and acutely lower blood pressure in hypertensive rats [36]. In the present study, the long-term blood pressure raising effects of SA were more likely due to its action on the kidney.

COX enzymatic products can have antihypertensive and prohypertensive properties, depending on the profile of prostanoids produced [37]. Long-term use of non-steroidal anti-inflammatory drugs and COX-2 inhibitors increased arterial blood pressure and induced edema and congestive heart failure in a significant proportion of patients [37]. SA inhibits COX, leading to increased metabolism of arachidonic acid via lipoxygenase (LOX) and cytochrome P450 (CYP) pathways. Products of the LOX pathway, 12- and 15-(S)-HPETE and 5- and 15-(S)-HETEs, can directly activate TRPV1 [38]. TRPV1 activation improves endothelial function, increases endothelium nitric oxide (NO) production, improves vasorelaxation, and may prevent hypertension [39]. Moreover, NO can also reduce RSNA [40], which might contribute to the increase in renal blood flow. However, SA treatment in TRPV1^−/−^ mice might lose these beneficial effects and cause further impairment on renal function.

SA suppressed urinary 6-keto PGF1α and PGE2 in obese WT and TRPV1^−/−^ mice, which might be related to a further increase in RSNA and elevation of blood pressure in obese TRPV1^−/−^ mice. COX-2 inhibitors prevent inflammatory response via inhibition of PG synthesis, which may also lead to blood pressure elevation, as PGE2 and PGI2 are important vasodilators. In response to WD feeding, the kidney increases synthesis of PGI2 and PGE2, which may be more pronounced in WD-fed TRPV1^−/−^ mice because TRPV1 ablation may decrease NO production [39] and other vasodilators such as calcitonin gene-related peptide (CGRP) and SP release. The present study showed that TRPV1 ablation increased urinary levels of 6-keto PGF1α, a major metabolite of PGI2 in obese TRPV1^−/−^ mice. PGI2 and PGE2 can activate and sensitize TRPV1 and increase SP and CGRP release [26,27], and these effects are absent in TRPV1^−/−^ mice. Taken together, SA treatment in obese TRPV1^−/−^ mice further decreased the release of vasodilators including SP, CGRP, PGE2, and PGI2, which might contribute to elevation of blood pressure and deterioration of renal function. SA-induced renal dysfunction in obese mice is dependent on whether the TRPV1 channels are intact.

Taken together, our data suggest that the inhibition of COX with SA in mice fed a WD diet increases ARNA and improves baroreflex control of sympathetic activity. SA-mediated nerve protective effects are abolished when TRPV1 is ablated, accompanied by exacerbated renal functional impairment and elevated blood pressure. These data may have significant clinical implications for obese and diabetic patients with impaired TRPV1 that may contribute to the development of resistant hypertension in these patients when treated with nonsteroidal anti-inflammatory drugs.

## Data Availability

All data are included in the paper.
